# Potential for alcohol and drug interactions in older adults: evidence from the Irish longitudinal study on ageing

**DOI:** 10.1186/1471-2318-14-57

**Published:** 2014-04-27

**Authors:** Gráinne Cousins, Rose Galvin, Michelle Flood, Mary-Claire Kennedy, Nicola Motterlini, Martin C Henman, Rose-Anne Kenny, Tom Fahey

**Affiliations:** 1 School of Pharmacy, Royal College of Surgeons in Ireland, Dublin 2, Ireland; 2 HRB Centre for Primary Care Research, Department of General Practice, Royal College of Surgeons in Ireland, Dublin 2, Ireland; 3 School of Pharmacy and Pharmaceutical Sciences, Trinity College Dublin, Dublin 2, Ireland; 4 The Irish Longitudinal Study of Ageing, Chemistry Extension, Trinity College Dublin, Dublin 2, Ireland

**Keywords:** Drug interactions, Alcohol interactive medications, Alcohol drinking/epidemiology, Aged

## Abstract

**Background:**

Older adults are susceptible to adverse effects from the concomitant use of prescription medications and alcohol. This study estimates the prevalence of exposure to alcohol interactive (AI) medications and concomitant alcohol use by therapeutic class in a large, nationally representative sample of older adults.

**Methods:**

Cross-sectional analysis of a population based sample of older Irish adults aged ≥60 years using data from The Irish Longitudinal Study on Ageing (TILDA) (N = 3,815). AI medications were identified using Stockley’s Drug Interactions, the British National Formulary and the Irish Medicines Formulary. An in-home inventory of medications was used to characterise AI drug exposure by therapeutic class. Self-reported alcohol use was classified as non-drinker, light/moderate and heavy drinking. Comorbidities known to be exacerbated by alcohol were also recorded (diabetes mellitus, hypertension, peptic ulcer disease, liver disease, depression, gout or breast cancer), as well as sociodemographic and health factors.

**Results:**

Seventy-two per cent of participants were exposed to AI medications, with greatest exposure to cardiovascular and CNS agents. Overall, 60% of participants exposed to AI medications reported concomitant alcohol use, compared with 69.5% of non-AI exposed people (p < 0.001). Almost 28% of those reporting anti-histamine use were identified as heavy drinkers. Similarly almost one in five, combined heavy drinking with anti-coagulants/anti-platelets and cardiovascular agents, with 16% combining heavy drinking with CNS agents. Multinomial logistic regression showed that being male, younger, urban dwelling, with higher levels of education and a history of smoking, were associated with an increased risk for concomitant exposure to alcohol consumption (both light/moderate and heavier) and AI medications. Current smokers and people with increasing co-morbidities were also at greatest risk for heavy drinking in combination with AI medications.

**Conclusions:**

The concurrent use of alcohol with AI medications, or with conditions known to be exacerbated by alcohol, is common among older Irish adults. Prescribers should be aware of potential interactions, and screen patients for alcohol use and provide warnings to minimize patient risk.

## Background

Older adults are susceptible to adverse effects from the concomitant use of prescription medications and alcohol, in part, because of changes in absorption, distribution and metabolism of alcohol and other medications with age [1]. In addition to these physiological changes older adults have reduced homeostatic capacity, and often take multiple alcohol interactive (AI) medications [2-4]. The major adverse clinical outcomes of medication-alcohol interactions include raising blood alcohol levels, altering the metabolism of drugs, liver toxicity, gastrointestinal inflammation and bleeding, sedation, disulfiram-like-reactions, and interference with the effectiveness of medications and exacerbating their adverse effects [5,6]. For example, alcohol potentiates the sedative effects of benzodiazepines, antidepressants (e.g. tricyclics), anithisamines, muscle relaxants and opioids [6], giving rise to falls, car accidents and death [7,8]. Nonsteroidal anti-inflammatory drugs (NSAIDs), when combined with alcohol, can increase an older patients risk for gastrointestinal bleeding [6]. A systematic review has also indicated that alcohol consumption exacerbates certain chronic conditions, such as liver disease, diabetes mellitus, gastritis and other gastrointestinal conditions, gout, depression, and breast cancer [9].

Although older adults typically report lower levels of heavy drinking [5,10,11], many continue to engage in patterns of alcohol consumption that exceed current guidelines for older adults [11-15]. Despite being susceptible to adverse effects from the concomitant use of AI medications and alcohol, few studies have considered the potential for alcohol-drug interactions in older adults [16,17]. One study found that 10% of older US adults were at risk for potential adverse effects due to concomitant use of alcohol and AI medications or conditions known to be exacerbated by alcohol, this estimate increased to 26% among current drinkers [18]. Another US study of community dwelling older adults identified 21.5% of current drinkers as at-risk drinkers due to their alcohol use combined with select comorbidities and 21.2% at-risk due to their alcohol use combined with AI medications [12]. While these studies provide prevalence estimates of the potential risk for adverse effects due to concomitant use, specific patterns of concomitant use are lacking [17]. Only one study to date has considered the prevalence of alcohol use by therapeutic class, with the most common combination of alcohol and AI medications occuring with NSAIDs (20.2%), antihistamines (20.1%) and anti-hypertensives (19.8%) [17]. These results relate to older US adults, which may differ from European countries, as Europe reports the highest per-capita alcohol consumption in the world with alcohol related deaths among older Europeans increasing considerably over the past ten years [19]. The purpose of this study is to estimate the prevalence of AI medication exposure and concomitant alcohol use by therapeutic class in a large, nationally representative population sample of older Irish adults. Additional objectives include: (i) the estimation of the prevalence of concomitant alcohol use with specific conditions known to deteriorate with alcohol consumption [9]; and (ii) the identification of sociodemographic and health factors associated with concomitant use of AI medications and alcohol.

## Methods

### Study population

This study was conducted in the context of The Irish LongituDinal Study on Ageing (TILDA). TILDA recruited a stratified clustered sample of 8,175 individuals, representative of the community dwelling population aged 50 years and older in Ireland between October 2009 and February 2011. Wave 1 of the TILDA study included a face-to-face interview in the participants’ home and a self-completion questionnaire, which was returned after the visit. The adjusted response rate to the study was 62% and response to the self-completion questionnaire was 84%. Full details of the study sample and response rates have been described elsewhere [20]. Home interviews were conducted by trained professional social interviewers using Computer Assisted Personal Interviewing (CAPI). Interviewers asked participants in their homes “to record all medications that you take on a regular basis, like every day or every week”, and to provide medication packages to copy down the correct medication names. Assistance from relatives was permitted. Medications were coded using the World Health Organization Anatomical Therapeutic Chemical (ATC) classification system.

Participants also completed a self-completion questionnaire which explored issues considered particularly sensitive for respondents to answer directly to an interviewer. The self-completed questionnaire included items relating to alcohol consumption and the CAGE screening tool for problem drinking [21]. Participants aged 60 years and older who responded to the alcohol items on the self-completion questionnaire are included in the analysis. The study was approved by the Faculty of Health Sciences Research Ethics Committee at Trinity College Dublin and all participants gave informed written consent.

### Measures

#### Alcohol consumption

Current drinkers were defined as individuals who reported drinking alcohol in the previous 6 months. The number of drinks consumed per day/ per week was calculated using self-reported quantity and frequency measures relating to the past six months. Using the standard quantity-frequency approach [22], participants were categorised as non-drinkers, light/moderate drinkers, or heavier drinkers. The National Institute on Alcohol Abuse and Alcoholism (NIAAA) developed age specific guidelines, advising limits of no more than three drinks per day or seven drinks per week for older men and women [23]. These limits are based on a standard drink in the US, i.e. any drink containing 14 grams of pure alcohol [23], and were were amended to four drinks per day or ten drinks per week given a standard drink in Ireland contains 10 grams of pure alcohol [24,25]. Using the NIAA as a guideline, the following thresholds were applied: heavier drinkers: in excess of NIAA limits, light/moderate drinkers: less than or up NIAA limits. Problem drinking was measured by the CAGE alcohol screening questionnaire consisting of four items detailing whether the respondent had ever 1) felt the need to cut down on their drinking, 2) been criticised by others due to excessive drinking, 3) felt bad or guilty due to drinking, or 4) had a drink in the morning to steady their nerves, get rid of a hangover or as an eye-opener. One point was allocated for each positive response. Problem drinking was defined as a score of ≥ 2. A cut point of ≥2 is associated with 71% sensitivity and 91% specificity for problem drinking in older adults [26].

### Use of AI medications

Prescribed and OTC drugs which have the potential to interact with alcohol were identified by Stockley’s Drug Interactions [27], the British National Formulary (BNF) [28] and the Irish Medicines Formulary (IMF) [29], as specified alcohol interactivity and/or provided a cautionary warning and/or recommendation for advisory labels. AI medications were classified according to nine therapeutic classes based on the BNF (cardiovascular agents; CNS agents; antihistamines; anti-coagulants/anti-platelets; antidiabetic agents; anti-infectives; gastrointestinal drugs; immunomodulators; and muslce relaxants).

Medical conditions known to be exacerbated by alcohol, as identified in a systematic review [9], were also recorded during the interview. Participants were asked if they ever received a diagnosis of diabetes mellitus, hypertension, peptic ulcer disease, liver disease or breast cancer from a doctor. Depressive symptoms, during the weeks before interview, were also measured by the 20-item Center for Epidemiologic Studies Depression (CES-D) scale [30]. Each symptom is scored on a scale from 0 (rarely or none of the time, less than one day) to 3 (most or all of the time, five-seven days) to give a possible total score of 60. Depression was defined as a score of ≥16. A cut point of ≥16 has 100% sensitivity and 88% specificity for major depression in community dwelling older adults [31]. Furthermore, prescription drugs for the treatment of gout were used as proxies for gout (ATC, M04AA01). A number of sociodemographic and health variables were also recorded, including age, gender, urbanicity, marital status, education, smoking status and self-reported health status.

### Data analysis

Statistical analyses were performed using STATA version 13. All analyses were weighted with respect to age, sex and education to ensure that data were nationally representative. Chi square analyses were used to examine the relationship between concomitant alcohol consumption and use of AI medications by therapeutic class, to determine whether use of AI medications in certain therapeutic classes had any effect on alcohol consumption. Additional analyses examined differences in drinking status by therapeutic class.

Multinomial logistic regression was used to identify factors associated with light/moderate, and heavier drinking in older adults using one or more AI medications. In this model, the outcome variable had 3 possible outcomes, with individuals taking at least one AI medication and not drinking alcohol (non-drinkers) as the reference category. Interpretation of results for a specific risk factor is based on the odds of being, for example, a heavier drinker using AI medications rather than a non-drinker using AI medications.

## Results

### Study population

3,815 adults over 60 years (mean age 69.7 ± 7.3, range 60–99 years) responded to alcohol items in the self-completed questionnaire, with 2,490 (62.8%) reporting alcohol consumption in the past 6 months. The prevalence of alcohol consumption declined with age, ranging from 78% (60–64 years), 70% (65–69 years), 59% (70–74 years), 55% (75–79 years) and 47% (80+ years). Using the NIAA guidelines, 37.2% (n = 1,325) were classified as non-drinkers; 43.1% (n = 1,708) as light to moderate drinkers; and 19.7% (782) were heavier drinkers (Table 1). The proportion of older adults reporting heavy drinking declined with age, however, one quarter of heavier drinkers were aged 65–69 with 17% aged 70–74 years.

**Table 1:** Characteristics of study population (N = 3,815)

**Characteristics**	**Nondrinker (n = 1,325)**	**Light-moderate drinker (n = 1,708)**	**Heavier drinker (n = 782)**	**Total (N = 3,815)**
**Gender**				
Male	494 (36.0)	714 (43.2)	566 (73.8)	1,774 (46.6)
Female	831 (64.0)	994 (56.8)	216 (26.2)	2,041 (53.4)
**Age**				
60-64	246 (19.5)	576 (36.2)	295 (42.9)	1,117 (31.3)
65-69	292 (19.6)	463 (24.3)	210 (24.5)	965 (22.6)
70-74	314 (21.2)	294 (15.6)	148 (16.8)	756 (17.9)
75-79	241 (19.7)	217 (13.8)	73 (9.0)	531 (15.0)
80+	231 (20.0)	155 (10.2)	55 (6.8)	441 (13.2)
**Residence**				
Rural	743 (58.3)	787 (48.1)	280 (37.4)	1,810 (49.7)
Urban	578 (41.8)	919 (52.0)	501 (62.6)	1998 (50.3)
**Marital status**				
Married	777 (56.1)	1,189 (69.5)	565 (71.3)	2,531 (64.9)
Widowed	350 (29.7)	309 (19.3)	116 (15.3)	775 (22.4)
Divorced/separated	46 (3.2)	66 (3.6)	49 (6.1)	161 (4.0)
Never married	152 (11.1)	144 (7.5)	52 (7.3)	348 (8.8)
**Education level**				
None/primary level	630 (58.3)	511 (39.1)	225 (37.1)	1,366 (45.9)
Secondary level	449 (31.9)	641 (40.9)	285 (40.4)	1,375 (37.4)
Third level	246 (9.8)	554 (20.0)	272 (22.5)	1,072 (16.7)
**Self-reported health status**				
Excellent to very good	687 (50.8)	994 (56.7)	439 (54.1)	2,120 (54.0)
Good	392 (29.7)	495 (29.6)	242 (31.9)	1,129 (30.1)
Fair to poor	241 (19.5)	217 (13.8)	101 (14)	559 (15.9)
**Depression (CES-D ≥16)**	109 (8.9)	116 (7.2)	50 (6.7)	275 (7.7)
**Smoking history**				
Current smoker	166 (13.2)	207 (12.8)	140 (19.6)	513 (14.3)
Past smoker	439 (33.2)	730 (43.2)	413 (51.1)	1,582 (41.0)
Never smoked	720 (53.6)	771 (44)	229 (29.3)	1,720 (44.7)

Note: Totals may not equal 100% because of rounding.

Problem drinking, defined by CAGE, was identified in 8% of the total sample (12.7% of current drinkers). The proportion of older adults with problem drinking declined with age, ranging from 12% (60–64 years), 9% (65–69 years), 7% (70–74 years), 4% (75–79 years) and 2% (80+ years). The prevalence of problem drinking was higher among men (12.2% vs 4%), living in an urban setting (10% vs 6%). Approximately, one in ten adults with third level education reported problem drinking (11%) compared to 7% of those with primary or secondary education. The prevalence of problem drinking was highest among participants who reported being separated or divorced (17.5%). This compares to approximately 9% among those who were married or never married and 5% of those widowed. A higher proportion of adults with depression (13% vs 8%), were identified as problem drinkers.

### Alcohol interactive medications and alcohol consumption

Table 2 shows AI drug exposure by therapeutic class. Almost three quarters (72%, n = 2,672) of all participants reported taking one or more AI medication, with a mean and standard deviation of 2.04 ± 2. Exposure varied by therapeutic class: cardiovascular agents (61.2%), CNS agents (22.6%) and blood anti-platelets (31%). Table 2 also shows the prevalence of alcohol use by therapeutic class. Sixty per cent of individuals using AI medications were drinkers compared to 69.5% of those not using AI medications (p <0.001). Individuals using AI medications in the following classes, cardiovascular, CNS, blood (anti-coagulant or anti-platelet) and antidiabetic, were significantly less likely to report drinking alcohol. Yet, more than half of those exposed to cardiovascular, CNS, blood or antidiabetic agents reported concurrent alcohol consumption. Table 2 also shows that the prevalence of alcohol use in combination with select conditions was high, particularly for those reporting gout (74.5%) and hypertension (65%).

**Table 2:** Alcohol-Interactive (AI) medication exposure, morbidity and prevalence of alcohol use by therapeutic class and morbidity (N = 3,815)

**Characteristics**	**Exposure to AI medications**	**Alcohol use in individuals using AI medications in class**	**Alcohol use in individuals *not *** **using AI medications in class**	**Total**
	**%**	**%**	**%**	**P-value**
**Cardiovascular agents**	*61.2*	*60.4*	*67.0*	*<0.001*
Angiotensin-receptor blockers & combinations	32.9	60.8	64.0	0.07
Beta blockers & combinations	19.5	57.5	64.3	0.001
Calcium channel blockers & combinations	13.7	57.8	63.8	0.01
Diuretics	12.5	53.5	64.3	<0.001
Alpha blockers	2.9	60.2	63.0	0.57
Vasodilator Antihypertensives	0.5	73.0	62.9	0.36
Nitrates	2.7	59.0	63.1	0.44
Cholesterol lowering agents	36.7	63.2	62.8	0.84
**CNS agents**	*22.6*	*53.5*	*65.7*	*<0.001*
NSAID	1.9	59.4	63.0	0.59
COX2 inhibitors	1.5	62.4	62.9	0.93
Opioid analgesics	3.1	51.8	63.3	0.02
Non-opioid analgesics	5.3	54.6	63.4	0.03
Anti-epileptics	2.8	50.2	63.3	0.008
Hypnotics	5.5	54.7	63.4	0.02
Anxiolytics	2.7	44.1	63.5	<0.001
Barbiturates	-			
Antipsychotics	0.9	43.2	63.1	0.03
Antidepressants	7.4	52.9	63.8	0.002
Stimulants	-	-	-	
Nausea and Vertigo	1.9	46.8	63.3	0.01
Antimigraine	-	-	-	
Antiparkinson drugs	0.6	57.6	63.0	0.60
Dopaminergics	0.18	84.8	62.9	0.25
Drugs for dementia	0.46			
Drugs for dependence	-			
**Antihistamines**	*0.9*	*66.9*	*62.9*	*0.65*
Sedating	0.13	63.0	63.0	0.99
Non-sedating	0.84	65.0	62.9	0.82
**Blood**	*34.2*	*58.5*	*65.3*	*<0.001*
Anti-coagulant	3.6	55.9	63.2	0.09
Anti-platelet	31.0	58.8	64.8	0.001
**Antidiabetic agents**	*7.2*	*53.8*	*63.7*	*0.002*
**Anti-infectives**	*0.2*	*47.0*	*63.0*	*0.43*
**Gastrointestinal drugs**	*1.2*	*49.5*	*63.1*	*0.09*
**Immunomodulators**	*1.0*	*50.7*	*63.1*	*0.10*
**Muscle relaxants**	*0.2*	*80.3*	*62.9*	*0.28*
**Other**	*3.0*	*69.6*	*62.8*	*0.18*
**Morbidity**	**Prevalence of condition**	**Alcohol use in individuals with condition**	**Alcohol use in individuals *without* condition**	
	**%**	**%**	**%**	**P-value**
Diabetes	9.5	52.5	63.9	< 0.001
Hypertension	47.7	65.0	67.3	0.22
Peptic ulcer	7.1	60.8	63.0	0.49
Gout	1.9	74.5	62.7	0.08
Depression(CES-D ≥16)	7.7	57.7	63.5	0.06
Liver disease	0.54	39.7	62.9	0.04
Breast cancer	2.3	63.8	62.8	0.86

**Figure 1:**
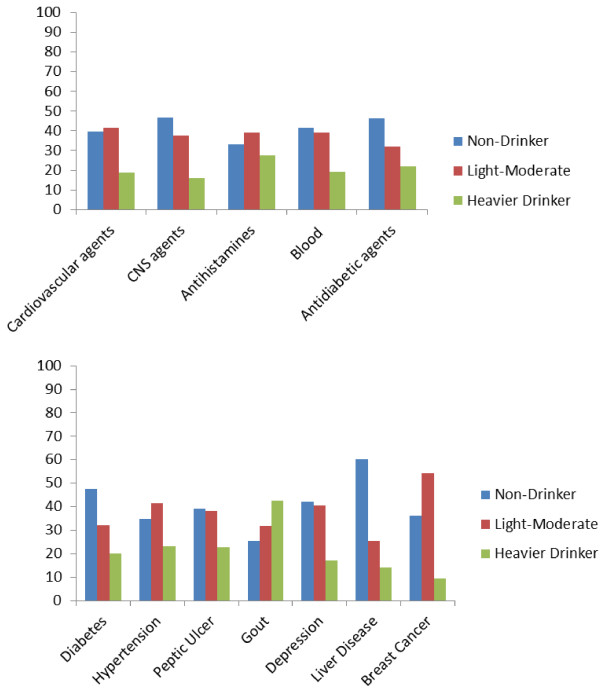
Drinking status by exposure to AI medications and morbidity (N = 3,815).

Further analysis of drinking status among AI drug users by therapeutic class are presented in Figure 1. More than a quarter of those reporting antihistamine use were identified as heavier drinkers. Similarly, almost one in five older adults combined heavy drinking with cardiovascular agents, blood (anti-coagulant or anti-platelet) and antidiabetic agents, with 16% combining heavy drinking with CNS agents. Thirteen per cent of people taking anti-epileptics, antipsychotics or hypnotics reported heavy drinking, with 18% of those taking antidepressants also reporting heavy drinking. Furthermore, approximately one in five older adults with diabetes, hypertension, peptic ulcer, or depression, were identified as heavy drinkers. Individuals with breast cancer were least likely to report heavy drinking (9.6%). In contrast, almost 43% of older adults with gout reported heavy drinking (Figure 1).

Results from the multinomial model are presented in Table 3. These results show consistency across a number of factors discriminating between various levels of concomitant use of alcohol with AI medications. Being male, younger, urban dwelling, better educated, never married and having a history of smoking distinguished drinkers at both levels from non-drinkers. Additional factors predicting heavy drinking in combination with AI medications included being a current smoker, and increasing co-morbidities. In contrast, increasing numbers of AI medications and fair to poor health were associated with a reduced odds of heavy drinking in combination with AI medications relative to non-drinkers.

**Table 3:** Results of multinomial logistic model of light-moderate, and heavier drinking combined with Alcohol Interactive (AI) medications (N = 2,672)

	**Light-Moderate drinker/nondrinker + AI**		**Heavier drinker/nondrinker + AI**	
**Characteristics**	**OR (95% CI)**	**P value**	**OR (95% CI)**	**P value**
**Gender**				
Male	1.00		1.00	
Female	0.75 (0.61 - 0.92)	0.006	0.18 (0.14 - 0.24)	<0.001
**Age**				
60-64	1.00		1.00	
65-69	0.80 (0.61 - 1.07)	0.13	0.64 (0.45 - 0.92)	0.02
70-74	0.46 (0.34 - 0.63)	<0.001	0.40 (0.27 - 0.59)	<0.001
75-79	0.50 (0 .36 - 0.69)	<0.001	0.25 (0.16 - 0.39)	<0.001
80+	0.38 (0.27 - 0.54)	<0.001	0.22 (0.13 - 0.35)	<0.001
**Residence**				
Rural	1.00		1.00	
Urban	1.48 (1.22 - 1.80)	<0.001	2.36 (1.80 - 3.08)	<0.001
**Marital status**				
Married	1.00		1.00	
Never married	0.49 (0.35 - 0.69)	<0.001	0.48 (0 .30 - 0.75)	0.001
Widowed	0.85 (0.66 - 1.10)	0.22	1.12(0.78 - 1.59)	0.54
Divorced/separated	0.57 (0.34 - 0.95)	0.03	0.83 (0.45 - 1.50)	0.53
**Education level**				
None/primary level	1.00		1.00	
Secondary level	1.41 (1.13 - 1.76)	0.002	1.91 (1.45 - 2.53)	<0.001
Third level	2.04 (1.60 - 2.61)	<0.001	3.07 (2.23 - 4.24)	<0.001
**Self-reported health status**				
Excellent to very good	1.00		1.00	
Good	1.12 (0.89 - 1.39)	0.33	1.12 (0.85 - 1.48)	0.42
Fair to poor	0.84 (0.63 - 1.12)	0.23	0.68 (0.46 - 0.98)	0.04
**Depression (CES-D** ≥16)	0.96 (0.79 - 1.16)	0.70	0.86 (0.68 - 1.09)	0.21
**Smoking history**				
Never smoked	1.00		1.00	
Current smoker	1.09 (0.81 - 1.47)	0.57	2.65 (1.79 – 3.93)	<0.001
Past smoker	1.52 (1.23- 1.89)	<0.001	2.07 (1.53 - 2.78)	<0.001
**Number of AI drugs**	0.95 (0.90 - 1.00)	0.09	0.91 (0.85 - 0.98)	0.018
**Number of co-morbidities**	1.04 (0.94 - 1.15)	0.49	1.29 (1.12 - 1.47)	<0.001

## Discussion

In this large, population-based sample of older people, 72% were exposed to at least one AI medication, with exposure varying by therapeutic class. Of those exposed to AI medications, 60% reported concomitant alcohol use, increasing their risk for alcohol related adverse reactions. This is in contrast to a large community based US study in adults of similar ages in which 20% of those on AI medications reported alcohol use [17]. The prevalence of alcohol use across each of the therapeutic classes was also larger in this study relative to the US study [17]. For example, we found that 53.5% of people using CNS agents concurrently drink alcohol, compared to 20.2% of older American adults. This may be due to a lower prevalence of self- reported alcohol consumption in the US study, 20.3% compared to 62.3% of older Irish adults. It is plausible that a higher proportion of older Irish adults drink alcohol, thereby increasing their potential risk for alcohol related adverse reactions. Furthermore, 8% of the current sample, (12.7% of current drinkers), were identified as problem drinkers on the CAGE screening questionnaire, which is higher than previous studies of older people [13,19]. Some of these differences may be explained by our younger cohort (60 years and older), however this is unlikely to account for the total variation as, although the prevalence of alcohol consumption declined with age, it remained relatively high among our older age-groups.

Although exposure to AI medications varied by therapeutic class, an examination of concurrent alcohol use suggests consistency across therapeutic classes. Concurrent alcohol use ranged between approximately 50-60% for most therapeutic classes, with the exception of muscle relaxants (80%). Similarly, heavy drinking was identified in approximately one in five older adults reporting concurrent use of cardiovascular, blood and anti-diabetic agents. The concurrent use of psychotropics with heavy alcohol consumption is of particular concern, as they may cause the most dangerous alcohol related adverse drug reactions [8]. Sixteen percent of older adults taking CNS agents were identified as heavy drinkers; 14.8% of people taking opioid analgesics reported heavy drinking. Similarly, 13% of people taking anti-epileptics, antipsychotics or hypnotics reported heavy drinking, with almost one in five of those taking antidepressants also reporting heavy drinking. This is consistent with other studies, which suggest that concurrent use of psychotropic drugs and alcohol has become more prevalent in older adults [32-34]. However, this may be an artefact of increased use of psychotropic medications with age. We also found a high prevalence of alcohol consumption among people with conditions known to be exacerbated by alcohol consumption.

Although we are unable to ascertain the reasons behind alcohol consumption, particularly heavy consumption, in combination with AI medications, previous research has shown that many older adults are often unaware of the potential risk [35]. This may be compounded by the fact that older people are less likely to reveal a history of excessive alcohol consumption [36], and healthcare workers have a lower degree of suspicion when assessing older people [37]. In light of these findings, general practitioners should take the opportunity to review their patients’ alcohol consumption prior to prescribing AI medications. Knowing how much alcohol their older patients are drinking would facilitate an effective assessment of risk, and identify those patients in need of counselling in relation to the safe use of alcohol and medications. Counselling patients on long-term AI medications is particularly important, as it may be easier to abstain from alcohol if concomitant medications are for defined short time periods compared to long-term medications. Pharmacists are also in a position to educate patients in relation to the potential risk associated with alcohol consumption and AI medication at the point of dispensing AI medications.

Limited clinican time for alcohol screening highlights the need to prioritise high-risk groups. Findings from the present study suggest that younger, better educated men, with a history of smoking and living in a city are at greatest risk. One positive finding was a 9% reduction in the odds of alcohol consumption among heavy drinkers with each additional AI drug taken. This is consistent with the US study [17]. In contrast, those with increasing numbers of co-morbitities, known to worsen with alcohol, were significantly more likely to engage in heavy drinking. This may represent a coping strategy.

When interpreting our findings, the following limitations should be considered. A number of medical conditions, and drinking frequency and quantity were based on self-report, which may result in misclassification bias. However, evidence suggests that self-reported alcohol consumption is both reliable and valid [38]. Furthermore, although this study identifies a number of risk factors that increase older peoples’ risk of exposure to alcohol-drug interactions, we do not have information on the drug strength, dose or duration or on the consequences or severity of these interactions. Further study of this cohort is necessary to quantify adverse outcomes associated with concomitant use of AI medications and alcohol.

Furthermore, the finding that alcohol consumption declines with age may represent a survival bias, with heavy drinkers either dying or stopping their alcohol at an earlier age. Future longitiudinal analysis is necessary to determine whether this age related decline remains with advancing age, as research has found that the average amount of alcohol consumed by older people who continue to drink does not change over time [39].

## Conclusions

In conclusion, these findings indicate that many Irish older adults drink in excess of the recommended limits for their age, with many using alcohol in combination with alcohol interactive medications or conditions known to be exacerbated with alcohol intake. As the proportion and age of the older population continues to increase, the absolute number using AI medications will also increase. This increase in the prevalence of AI medications, combined with increased use of alcohol suggests the proportion of older adults at risk of alcohol related adverse drug events will increase in future years [17]. These findings highlight the importance of patient education to ensure older adults understand the potential risks associated with their combined alcohol and medication use. Prescribers should also be aware of the potential interactions, and screen patients for alcohol use and provide warnings to minimize patient risk.

## Competing interests

No competing interests declared.

## Authors’ contributions

All authors were involved in the study conception and design. MF, MCK and MCH identified the alcohol interactive medications. GC and NM completed the statistical analysis. GC and RG interpreted the data and drafted the paper. TF, RK, MF, MCK and MCH provided methodological and content expertise during the manuscript preparation and critically revised the draft manuscript. All authors read and approved the final manuscript.

## Pre-publication history

The pre-publication history for this paper can be accessed here:

http://www.biomedcentral.com/1471-2318/14/57/prepub
